# A Newly Developed Anti-L1CAM Monoclonal Antibody Targets Small Cell Lung Carcinoma Cells

**DOI:** 10.3390/ijms25168748

**Published:** 2024-08-11

**Authors:** Miki Yamaguchi, Sachie Hirai, Masashi Idogawa, Toshiyuki Sumi, Hiroaki Uchida, Yuji Sakuma

**Affiliations:** 1 Department of Molecular Medicine, Research Institute for Immunology, Sapporo Medical University School of Medicine, Sapporo 060-8556, Japan; mikiyama@sapmed.ac.jp (M.Y.); hirai@sapmed.ac.jp (S.H.); tsh715@gmail.com (T.S.); 2 Department of Medical Genome Sciences, Cancer Research Institute, Sapporo Medical University School of Medicine, Sapporo 060-8556, Japan; idogawa@sapmed.ac.jp; 3 Department of Pulmonary Medicine, Hakodate Goryoukaku Hospital, Hakodate 040-8611, Japan; 4 Laboratory of Oncology, School of Life Sciences, Tokyo University of Pharmacy and Life Sciences, Tokyo 192-0392, Japan; hiuchida-tky@umin.net

**Keywords:** small cell lung cancer (SCLC), L1 cell adhesion molecule (L1CAM), antibody–drug conjugate (ADC), NEUROD1, ASCL1

## Abstract

Few effective treatments are available for small cell lung cancer (SCLC), indicating the need to explore new therapeutic options. Here, we focus on an antibody–drug conjugate (ADC) targeting the L1 cell adhesion molecule (L1CAM). Several publicly available databases reveal that (1) L1CAM is expressed at higher levels in SCLC cell lines and tissues than in those of lung adenocarcinoma and (2) the expression levels of L1CAM are slightly higher in SCLC tissues than in adjacent normal tissues. We conducted a series of in vitro experiments using an anti-L1CAM monoclonal antibody (termed HSL175, developed in-house) and the recombinant protein DT3C, which consists of diphtheria toxin lacking the receptor-binding domain but containing the C1, C2, and C3 domains of streptococcal protein G. Our HSL175-DT3C conjugates theoretically kill cells only when the conjugates are internalized by the target (L1CAM-positive) cells through antigen–antibody interaction. The conjugates (an ADC analog) were effective against two SCLC-N (NEUROD1 dominant) cell lines, Lu-135 and STC-1, resulting in decreased viability. In addition, L1CAM silencing rendered the two cell lines resistant to HSL175-DT3C conjugates. These findings suggest that an ADC consisting of a humanized monoclonal antibody based on HSL175 and a potent anticancer drug would be effective against SCLC-N cells.

## 1. Introduction

Patients with non-small cell lung carcinoma (NSCLC) with driver mutations have benefited from efficacious targeted treatments such as tyrosine kinase inhibitors (TKIs), resulting in dramatically improved survival. In contrast, immune checkpoint inhibitors are effective for those who lack such genetic alterations. However, TKIs are still unavailable for patients with small cell lung carcinoma (SCLC), and even immune checkpoint inhibitors are much less effective against SCLC [1,2], thereby necessitating novel therapeutic options. 

This study explored the possibility of antibody–drug conjugates (ADCs), which we believe would show high efficacy against SCLC. ADCs are developed by conjugating an anticancer agent to a monoclonal antibody (mAb). The mAbs for ADCs need to be internalized by target cells upon binding to their target membranous proteins [3]. We previously developed an efficient screening method for selecting such mAbs [4] and then developed several new mAbs against NSCLC or SCLC [5,6,7]. The critical component of our screening system is a recombinant protein termed DT3C, consisting of diphtheria toxin (DT) lacking the receptor-binding domain but containing the C1, C2, and C3 domains of streptococcal protein G (3C) [4]. Consequently, a mAb-DT3C conjugate can kill cancer cells only when the mAb being examined is internalized by the target cells through an antigen–mAb interaction [4]. 

Here, we focus on the L1 cell adhesion molecule (L1CAM) as a potential therapeutic target for SCLC. L1CAM was initially described as a critical molecule in the development and plasticity of the central nervous system. It facilitates neuronal migration, axon guidance, myelination, and synaptogenesis [8]. Furthermore, it has been reported that L1CAM enhances the invasion and metastasis of cancer cells in several types of cancer, and its elevated expression seems to correlate with shorter survival in patients with cancer, including endometrial cancer, ovarian cancer, and NSCLC [8,9]. However, as far as we know, no articles have clarified the expression or roles of L1CAM in SCLC except for a report using L1CAM as a neuronal differentiation marker [10]. Although SCLC was once regarded as a homogeneous disease, it is now considered to comprise four subtypes—SCLC-A (ASCL1 dominant), -N (NEUROD1 dominant), -P (POU2F3 dominant), and -Y (YAP1 dominant) subtypes [11,12].

In this study, we explored whether L1CAM could be a suitable therapeutic target in SCLC. We demonstrated the following: (1) L1CAM is highly expressed in SCLC cell lines and tissues compared with lung adenocarcinoma (LUAD); (2) its expression is notable in the SCLC-N subtype [11,12]; (3) we have developed a novel anti-human L1CAM mAb, HSL175; and (4) HSL175-DT3C conjugates can induce SCLC-N cells to undergo apoptosis. 

## 2. Results

### 2.1. L1CAM mRNA Is Highly Expressed in SCLC Cell Lines and Tissues

**Figure 1:**
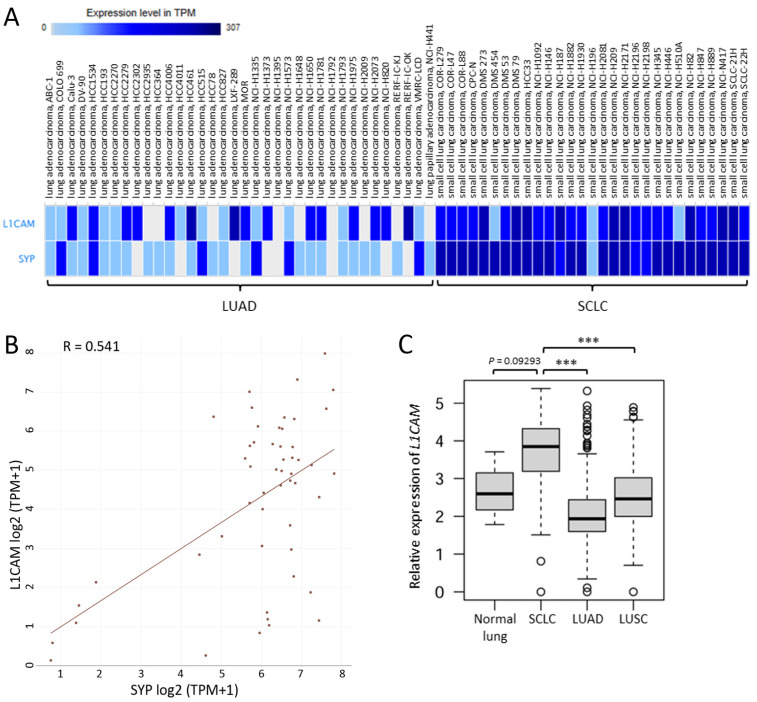
Small cell lung carcinoma (SCLC) cell lines and tissues express *L1CAM* mRNA. (**A**) *L1CAM* and *SYP* mRNA expression in SCLC and lung adenocarcinoma (LUAD) cell lines. The RNA-seq data of 29 SCLC and 36 LUAD cell lines are presented. (**B**) Correlation between *L1CAM* and *SYP* mRNA expression in SCLC cell lines (*n* = 52). Pearson’s correlation coefficient (R) = 0.541. (**C**) *L1CAM* mRNA expression in normal lung, SCLC, LUAD, and LUSC tissues. Expression data from 7 normal lung and 79 SCLC tissues were obtained from a previous report [13]. Expression data from 534 LUAD and 502 LUSC samples were derived from the Cancer Genome Atlas database [14,15]. *** *p* < 0.001.

Compared with LUAD cell lines, most of the SCLC cell lines examined expressed high levels of *L1CAM* as well as *SYP* mRNA, a neuroendocrine marker [11] (Figure 1A). As expected, these two genes were well correlated in expression with each other (Figure 1B; Pearson’s correlation coefficient: 0.541). Notably, *L1CAM* mRNA was expressed to a significantly greater extent in SCLC tissues than in LUAD or lung squamous cell carcinoma (LUSC) tissues (Figure 1C). Although the difference did not reach statistical significance, the expression levels of *L1CAM* mRNA in SCLC tissues were slightly higher than those in adjacent normal tissues (Figure 1C).

### 2.2. Two SCLC-N Cell Lines (Lu-135 and STC-1) Express L1CAM

**Figure 2:**
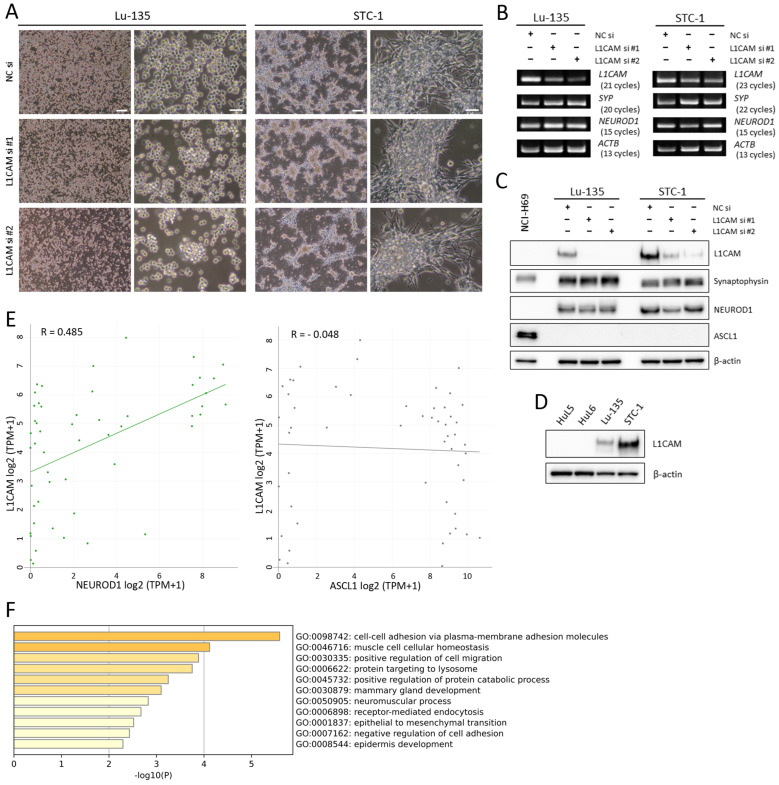
Two SCLC cell lines (Lu-135 and STC-1) express L1CAM. (**A**) Phase contrast images of Lu-135 and STC-1 cells with L1CAM silencing. Cells were reverse-transfected with NC siRNA, L1CAM siRNA #1, or L1CAM siRNA #2 (10 nM each) and cultured for 48 h. Scale bars: 200 μm (left; low magnification) and 50 μm (right; high magnification). (**B**) Conventional RT-PCR for the expression of *L1CAM*, *SYP*, *NEUROD1*, and *ACTB* mRNA in Lu-135 and STC-1 cells. Cells were treated as described in (**A**). (**C**) Western blot analysis of the cells treated as described in (**A**). Of note, NCI-H69 was used as a positive control for ASCL1. (**D**) Western blot analysis of HuL cells and SCLC-N cells for L1CAM. (**E**) Correlation between *L1CAM* and *NEUROD1* or *ASCL1* mRNA expression in SCLC cell lines (*n* = 52). (**F**) Regulated Gene Ontology results for the top 100 protein-coding DEGs in L1CAM-silenced Lu-135 cells compared with control cells.

Although the two SCLC cell lines (Lu-135 and STC-1) extensively examined in the present study were different in morphology, they expressed NEUROD1 (but not ASCL1) similarly. Therefore, they are classified as SCLC-N based on the current SCLC molecular subtypes [11,12] (Figure 2A–C). The two cell lines clearly expressed L1CAM at the mRNA and protein levels, while both siRNAs, especially siRNA #2, effectively suppressed the expression (Figure 2B,C). In contrast to SCLC-N cells, L1CAM protein was not observed in human lung epithelial cells (HuL5 or HuL6), derived from normal, peripheral lung tissues [16] (Figure 2D). As shown in Figure 2C, NCI-H69 cells (an SCLC-A cell line) were negative for L1CAM. Moreover, *L1CAM* mRNA expression was positively correlated with that of *NEUROD1*. However, such a correlation was not observed between *L1CAM* and *ASCL1* mRNA (Figure 2E). An enrichment analysis of the top 100 protein-coding differentially expressed genes (DEGs) suggested that spheroid formation through enhanced cell–cell adhesion was induced at least in Lu-135 cells when L1CAM was silenced (Figure 2A,F).

### 2.3. Lu-135 Cells Are Highly Sensitive to HSL175-DT3C Conjugates

**Figure 3:**
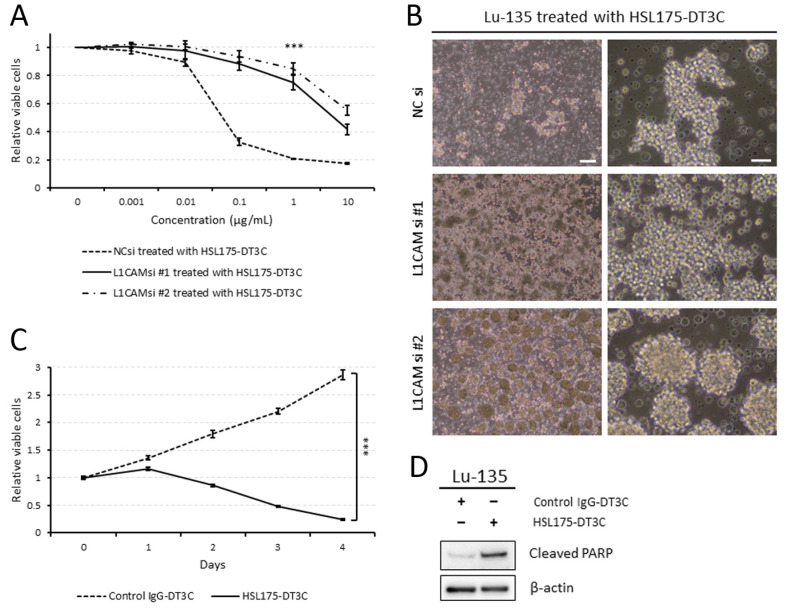
Lu-135 cells are highly sensitive to HSL175-DT3C conjugates. (**A**) Effects of HSL175-DT3C conjugates on the viability of Lu-135 cells. Cells were transfected with NC siRNA, L1CAM siRNA #1, or L1CAM siRNA #2 (10 nM each), cultured for 48 h, and then incubated with HSL175-DT3C conjugates (0–10 μg/mL each) for another 72 h. Results are presented as mean ± SD. *** *p* < 0.001. (**B**) Phase contrast images of Lu-135 cells. Cells were transfected with NC siRNA, L1CAM siRNA #1, or L1CAM siRNA #2 (10 nM each), cultured for 48 h, and then incubated with HSL175-DT3C conjugates (1 μg/mL each) for another 72 h. Scale bars: 200 μm (left; low magnification) and 50 μm (right; high magnification). (**C**) Effects of HSL175-DT3C conjugates on the viability of Lu-135 cells. Cells were cultured with control IgG-DT3C conjugates or HSL175-DT3C conjugates (0.1 μg/mL each) for 96 h. Results are presented as mean ± SD. *** *p* < 0.001. (**D**) Effects of HSL175-DT3C conjugates on levels of the apoptosis marker cleaved PARP in Lu-135 cells. Cells were cultured with control IgG-DT3C conjugates or HSL175-DT3C conjugates (1 μg/mL each) for 72 h.

When treated with HSL175-DT3C conjugates, NC siRNA-transfected (control) Lu-135 cells declined in viability in a concentration-dependent manner, whereas L1CAM-silenced cells became significantly less sensitive to the conjugates (Figure 3A,B). Figure 3B also illustrates that L1CAM knockdown, especially when using siRNA #2, induced Lu-135 cells to form spheroids, as evidenced by the GO enrichment analysis (Figure 2F). As presented in Figure 3C, the viability of Lu-135 cells decreased in a time-dependent manner when treated with HSL175-DT3C conjugates. Unlike the control IgG-DT3C conjugates, HSL175-DT3C conjugates effectively prompted Lu-135 cells to undergo apoptosis (Figure 3D).

### 2.4. HSL175-DT3C Conjugates Are Effective against STC-1 Cells

**Figure 4:**
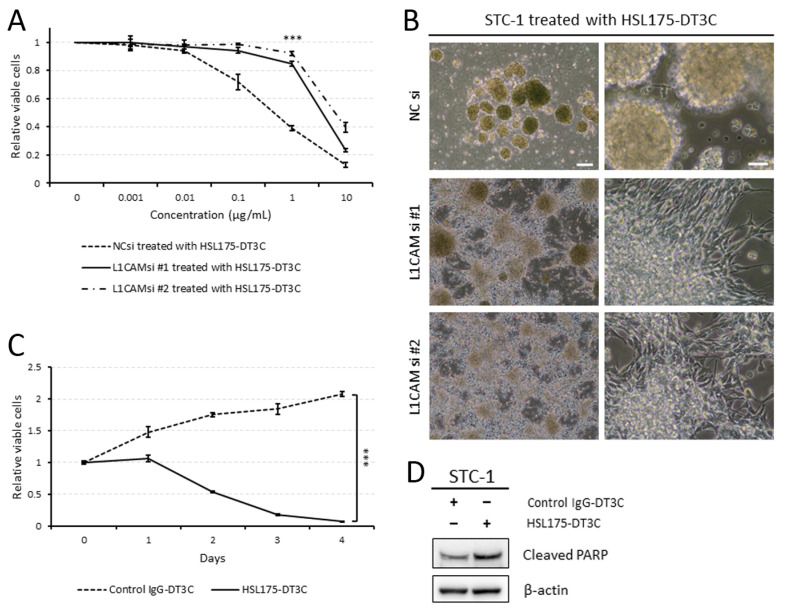
HSL175-DT3C conjugates are also effective against STC-1 cells. (**A**) Effects of HSL175-DT3C conjugates on the viability of STC-1 cells. Cells were transfected with NC siRNA, L1CAM siRNA #1, or L1CAM siRNA #2 (10 nM each), cultured for 48 h, and then incubated with HSL175-DT3C conjugates (0–10 μg/mL each) for another 72 h. Results are presented as mean ± SD. *** *p* < 0.001. (**B**) Phase contrast images of STC-1 cells. Cells were transfected with NC siRNA, L1CAM siRNA #1, or L1CAM siRNA #2 (10 nM each), cultured for 48 h, and then incubated with HSL175-DT3C conjugates (1 μg/mL each) for another 48 h. Scale bars: 200 μm (left; low magnification) and 50 μm (right; high magnification). (**C**) Effects of HSL175-DT3C conjugates on the viability of STC-1 cells. Cells were cultured with control IgG-DT3C conjugates or HSL175-DT3C conjugates (1 μg/mL each) for 96 h. Results are presented as mean ± SD. *** *p* < 0.001. (**D**) Effects of HSL175-DT3C conjugates on levels of the apoptosis marker cleaved PARP in STC-1 cells. Cells were cultured with control IgG-DT3C conjugates or HSL175-DT3C conjugates (1 μg/mL each) for 48 h.

As shown in Figure 4A–C, STC-1 cells were also sensitive to HSL175-DT3C conjugates, leading to reduced viability in a concentration- and time-dependent manner. Of note, Figure 4B demonstrates that control STC-1 cells were not able to adhere to the plate whereas L1CAM-silenced cells remained adherent to the dishes in the presence of HSL175-DT3C conjugates. Furthermore, STC-1 cells treated with HSL175-DT3C conjugates underwent apoptosis to a greater extent than those treated with control IgG-DT3C conjugates (Figure 4D).

## 3. Discussion

In this study, we have shown that (1) *L1CAM* mRNA is highly expressed in SCLC cell lines and tissues; (2) its expression correlates with that of *NEUROD1* but not *ASCL1* mRNA in SCLC cell lines; (3) a newly developed anti-human L1CAM mAb, HSL175, is internalized by L1CAM-positive cells upon binding; and (4) HSL175-DT3C conjugates cause SCLC-N cells to undergo apoptosis in a dose- and time-dependent manner. Although L1CAM (also known as neural cell adhesion molecule-like 1) was initially identified as a critical molecule in the development and plasticity of the central nervous system, it has been noted that it enhances invasion, metastasis, and chemoresistance in several types of cancer, including glioma and endometrial cancer [8,9]. L1CAM is likely to help maintain stemness of glioma, colorectal cancer, and ovarian cancer stem cells [17,18,19]. As a result, anti-L1CAM neutralizing mAbs or CAR-T cells targeting L1CAM have already been developed, while mAbs that can be utilized for ADCs have not been reported thus far [8]. Therefore, this is the first report that describes the development of anti-L1CAM mAbs for ADCs. Experimental findings in the present study suggest that an ADC consisting of a humanized mAb based on HSL175 and a potent anticancer drug could be highly effective against SCLC-N cells. We also found that HuL cells, a normal epithelial stem cell in the peripheral lung [16], were negative for L1CAM unlike SCLC-N cells (Figure 2D).

As mentioned above, L1CAM appears to act as an oncogene in many kinds of cancer [8]. It has also been reported to be a tumor suppressor gene in pancreatic ductal adenocarcinoma [20]. L1CAM expression levels in pancreatic cancer tissues appear to be lower than in surrounding normal tissues [20]. Since SCLC tissues expressed *L1CAM* mRNA to a greater extent than adjacent normal tissues (Figure 1C), it is likely to act as an oncogene in SCLC like glioma or gynecological cancer. However, L1CAM knockdown seems to enhance spheroid formation in Lu-135 cells (Figure 2F and Figure 3B). Thus, we cannot exclude the possibility that L1CAM silencing promotes the stemness of SCLC cells. Alternatively, it may function as a tumor suppressor gene in SCLC. Much remains to be studied on the role of L1CAM in SCLC.

There are several critical limitations in this study. The KD of the anti-human L1CAM mouse mAb (HSL175) and of the HSL175-DT3C conjugate was not determined. Future studies to evaluate the KD need to be conducted to better understand the potential of an ADC consisting of a humanized mAb based on HSL175 and a potent anticancer drug. Also, the number of SCLC-N cell lines examined was very limited. Moreover, we only conducted in vitro experiments in this study. Many more SCLC cell lines remain to be analyzed in vivo as well as in vitro. This is partly because L1CAM is also expressed in non-cancerous cells [8]. Additional preclinical in vitro and in vivo studies remain be performed to evaluate the toxicity of anti-L1CAM mAb-based drugs. 

In this work, we have developed an anti-L1CAM mAb, HSL175, that is internalized by L1CAM-expressing cells upon binding. Consequently, our HSL175-DT3C conjugates (an ADC analog) decrease the viability of SCLC-N cells in a dose- and time-dependent manner. These results raise the possibility that an L1CAM-targetig ADC would be effective against SCLC-N cells, which is more invasive than the other SCLC subtypes [21]. 

## 4. Materials and Methods

### 4.1. The Expression Atlas

In order to clarify the extent to which SCLC and LUAD cell lines expressed *L1CAM* and synaptophysin (*SYP*) mRNA, a reliable neuroendocrine differentiation marker [11,12], we utilized the RNA-sequencing (RNA-seq) data of 675 commonly used human cancer cell lines [22] in the Expression Atlas (https://www.ebi.ac.uk/gxa/experiments/E-MTAB-2706/Results; accessed on 31 March 2024). 

### 4.2. The Cancer Dependency Map

The Cancer Dependency Map (https://depmap.org/portal/; accessed on 31 March 2024) was used to elucidate to what degree *L1CAM* mRNA expression was positively correlated with *SYP*, *NEUROD1*, and *ASCL1* mRNA expression in SCLC cell lines [23]. 

### 4.3. Comparison of L1CAM mRNA Expression in Normal Lung and Lung Cancer Tissues

We used the RNA-seq data of normal lung (*n* = 7) and SCLC (*n* = 79) tissues provided by Jiang et al. [13] and LUAD (*n* = 534) and LUSC (*n* = 502) samples obtained from the Cancer Genome Atlas database [14,15] to analyze the expression levels of *L1CAM* mRNA in normal lung, SCLC, LUAD, and LUSC tissues, as described previously [7]. Expression levels for *L1CAM* mRNA are illustrated in a box-and-whisker plot. 

### 4.4. Cell Culture

The two SCLC cell lines (Lu-135 and STC-1) examined in this study were obtained from the Japanese Cancer Research Resources Bank (JCRB; Osaka, Japan) and cultured in RPMI-1640 medium (FUJIFILM Wako Pure Chemical, Osaka, Japan) containing 10% (*v*/*v*) fetal bovine serum, ultra-low IgG (Hyclone, Thermo Fisher Scientific Japan, Yokohama, Japan) and antibiotics at 37 °C in a humidified incubator with 20% O_2_ + 5% CO_2_. Moreover, human lung epithelial cells (HuL cells), derived from normal, peripheral lung tissues, were isolated as described previously [16]. HuL5 and HuL6 cells were maintained in bronchial epithelial cell growth medium (BEGM, Lonza Japan, Tokyo, Japan), a serum-free media, containing EW-7197, the potent TGF-β receptor inhibitor (1 μM, Selleck Chemicals, Houston, TX, USA), and Y-27632, the selective Rho-associated coiled-coil containing protein kinase 1 and 2 inhibitor (10 μM, Cayman Chemical, Ann Arbor, MI, USA) at 37 °C in a humidified incubator with 20% O_2_ + 5% CO_2_ [16]. 

### 4.5. RNA Interference (RNAi) Assay

For the transient knockdown of L1CAM, 1 × 10^6^ cells were plated in 60 mm culture dishes and transfected with negative control (NC) small interfering RNA (siRNA) duplexes (1027281; Qiagen, Hilden, Germany) or siRNA duplexes targeting L1CAM using Lipofectamine RNAiMAX reagent and OPTI-MEM I (Thermo Fisher Scientific) as described previously [6,7]. In this study, we purchased two different siRNA duplexes: Silencer Select Pre-Designed siRNAs s536516 and s536517 (termed L1CAM siRNA #1 and #2, respectively; Thermo Fisher Scientific). The final concentration of siRNA used in each in vitro experiment was 10 nM. The downregulation of targeted gene expression was verified by reverse transcription PCR (RT-PCR) and Western blot. 

### 4.6. RNA Isolation and RT-PCR

Total RNA extraction and conventional RT-PCR were conducted as previously described [6,7]. The following PCR primers (QuantiTect Primer Assay) were purchased from Qiagen: *L1CAM* (Hs_L1CAM_1_SG), *SYP* (Hs_SYP_1_SG), *NEUROD1* (Hs_NEUROD1_1_SG), and *ACTB* (Hs_ACTB_1_SG). The amount of *ACTB* mRNA in each sample was used to standardize the quantity of target mRNAs. 

### 4.7. Western Blot Analysis

Cultured cells were lysed in NuPAGE LDS Sample Buffer (Thermo Fisher Scientific). Whole cell lysates were subjected to SDS-PAGE (5–20% SuperSep Ace; FUJIFILM Wako Pure Chemical) followed by blotting with specific antibodies and detection using the Supersignal West Pico Plus Chemiluminescent Substrate (Thermo Fisher Scientific) as described previously [6]. The primary antibodies used were anti-L1CAM (#90269; clone D5D3K; 1:1000; Cell Signaling Technology), anti-synaptophysin (#36406; clone D8F6H; 1:1000; Cell Signaling Technology), anti-NEUROD1 (#7019; clone D90G12; 1:1000; Cell Signaling Technology), anti-ASCL1 (#10585; clone E5S4Q; 1:1000; Cell Signaling Technology), anti-cleaved poly (ADP-ribose) polymerase (PARP) (Asp214) (#5625; clone D64E10; 1:1000; Cell Signaling Technology), and anti-β-actin (A1978; clone AC-15; 1:10,000; Sigma-Aldrich Japan, Tokyo, Japan). 

### 4.8. Development of a Novel Anti-Human L1CAM Mouse mAb (HSL175)

We carried out all animal experimentation in accordance with a protocol approved by the Animal Committee of Sapporo Medical University (approval code: 21-066; approval date: 16 August 2021). The Lu-135 cells, which express L1CAM, were used to immunize a Balb/c mouse using procedures described previously [4,5,6,7]. Briefly, two days after the final injection of Lu-135 cells, the mouse was sacrificed and 7 × 10^7^ splenocytes were fused with 5 × 10^5^ P3U1 cells with polyethylene glycol. When the hybridomas had grown to ~50% confluence, the culture supernatant fluid, which contained polyclonal antibodies, was tested for antibody internalization with DT3C. We ultimately gained an anti-human L1CAM mouse mAb (IgG3, κ), named HSL175, that was internalized by L1CAM-expressing SCLC cells upon binding. 

### 4.9. Evaluating the Efficacy of HSL175 mAb-DT3C Conjugates

HSL175 mAb or control IgG were incubated with DT3C at room temperature for 30 min to generate mAb-DT3C conjugates [4,5,6,7]. The control IgG used in this experiment was mouse IgG3, κ isotype control antibody (clone MG3-35; BioLegend, San Diego, CA, USA). In theory, each conjugate comprised one mAb (~150 kDa) and two DT3C molecules (~140 kDa) [4,5,6,7]. Two SCLC cell lines (Lu-135 and STC-1) were seeded and incubated in the presence of HSL175-DT3C or control IgG-DT3C conjugates (0–10 μg/mL each) for 48–96 h. 

### 4.10. Assessment of Cell Viability and Apoptosis

As previously described, the cell viability was evaluated using a CellTiter Glo 3D Cell Viability Assay (Promega, Madison, WI, USA) [6,7]. All results are presented as mean ± standard deviation (SD). Apoptosis was assessed by Western blot analysis of cleaved PARP as described previously [6,7]. 

### 4.11. Differentially Expressed Genes (DEGs) in L1CAM-Silenced Lu-135 Cells 

Lu-135 cells transfected with NC siRNA, L1CAM siRNA #1, or L1CAM siRNA #2 were cultured for 48 h, and total RNA was then isolated using an RNeasy Mini Kit (Qiagen). An RNA-seq analysis was conducted by Novogene (Queenstown, Singapore). A total of 323 DEGs, including 138 upregulated and 185 downregulated genes, were identified between two separate analyses (L1CAM siRNA #1-transfected cells vs. controls and L1CAM siRNA #2-transfected cells vs. controls); the DEGs are listed in Supplementary Table S1. We finally used Metascape to carry out a Gene Ontology enrichment analysis of the top 100 protein-coding DEGs [24].

### 4.12. Statistical Analysis

A two-tailed Student’s *t*-test or one-way analysis of variance followed by the Tukey–Kramer multiple comparisons test was conducted to evaluate differences in cell viability between cells differentially treated in vitro. With regard to *L1CAM* mRNA expression in normal lung, SCLC, LUAD, and LUSC tissues, *p*-values were calculated by Welch’s two-sided *t*-test. *p* < 0.05 denotes statistical significance. All statistical calculations were performed using JMP Pro software version 15 (SAS Institute Japan, Tokyo, Japan). 

## Data Availability

Data are contained within the article and Supplementary Materials.

## Supplementary Materials

The following supporting information can be downloaded at: https://www.mdpi.com/article/10.3390/ijms25168748/s1.
